# Encephalitis in a child with H1N1 infection: First case report from India

**DOI:** 10.4103/1817-1745.76119

**Published:** 2010

**Authors:** Rajesh Kulkarni, Aarti Kinikar

**Affiliations:** Department of Pediatrics, B. J. Medical College, Pune, India

**Keywords:** Encephalitis, H1N1

## Abstract

Neurological complications have been described with seasonal influenza infection. We report encephalitis manifesting as seizures in a child with confirmed H1N1 infection. Treatment with oseltamivir was started. Child was discharged without any neurological sequelae.

## Introduction

There are only few case reports describing the association of novel influenza A (H1N1) virus with encephalopathy or encephalitis in children.[1,2] We report a previously well child with confirmed H1N1 infection who presented with seizures, CSF pleocytosis, abnormal EEG and focal changes on CT brain.

## Case Report

A previously healthy eight-year-old boy was brought to our institute during the current H1N1 pandemic with moderate fever, cough and occipital headache since five days and one episode of generalized tonic clonic convulsion, associated with fever, just before admission. Immediately after admission, he developed right-sided focal convulsions which were controlled with intravenous midazolam.

On admission, the patient was afebrile with a blood pressure of 110/70 mmHg, a heart rate of 82 beats per minute and a respiratory rate of 24 per minute. Oxygen saturation measured by pulse oximetry was 97% on room air, and a rapid assay for glucose was 96 mg/dl. Child was drowsy with a GCS scale of 10/15. Pupils were 2 mm bilaterally and reactive. Terminal neck stiffness was present. Rest of the CNS examination was normal.

**Figure 1:**
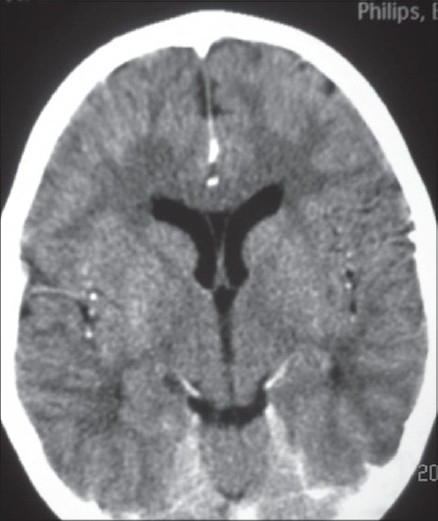
CT brain showing ill-defi ned hypodensities in subcortical white matter in both parietal lobes.

**Figure 2:**
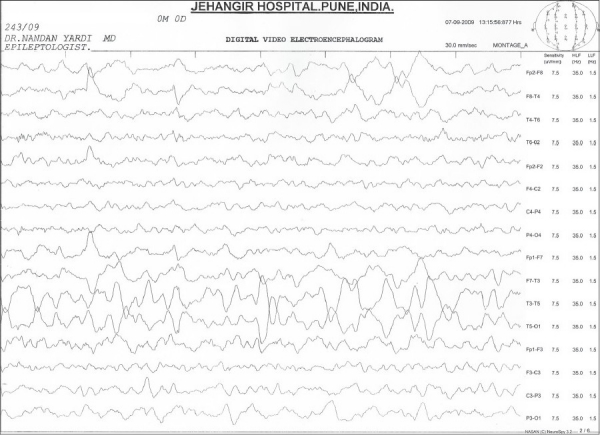
EEG showing focal slowing and sharp activity from the left centroparietal region.

Routine hemogram, blood gas and serum electrolytes were normal. A CT and lumbar puncture were performed in view of focal seizures and no improvement of sensorium after 4 hours of admission. CT was performed before lumbar puncture and showed ill-defined hypodensities (CT value 16-23 HU) in the subcortical white matter in the posterior parietal lobes bilaterally suggestive of encephalitis [Figure 1]. Lumbar puncture showed 40 cells/mm^3^, proteins were 48 mg/dl and glucose was 71 mg/dl with no organisms seen on gram stain. A chest X-ray revealed a small focal consolidation in the right lower zone. Nasopharyngeal aspirate specimen tested for H1N1 by RT-PCR tested positive. CSF was negative for H1N1 (by RT-PCR). CSF PCR for Herpes simplex virus was negative. Additional viruses such as enterovirus, varicella zoster, cytomegalovirus and Epstein-Barr virus tests could not be done due to financial constraints. Broad spectrum IV antibiotic, oseltamivir and acyclovir treatment were initiated. EEG showed evidence of focal slowing and sharp activity from the left centroparietal region [Figure 2]. The sensorium improved after 48 h and there were no further seizures. The patient was given oseltamivir for five days. He was discharged after 10 days of hospitalization.

## Discussion

The incidence of seasonal influenza-related neurologic complications has been estimated at 4 cases per 100,000 children per year.[3] Neurologic complications appear more commonly in children under six years of age.[4] Neurologic manifestations include seizures, loss of consciousness, cranial nerve abnormalities, focal motor deficits and gait abnormalities, among others.[4]

The pathogenesis of influenza-associated neurologic disease remains unclear. Influenza RNA is rarely detected in the CSF of encephalopathic patients.[5] Other possible hypothesis is systemic immune response. High levels of proinflammatory cytokines (like IL-6 or TNF alpha) have been demonstrated in the serum and in the cerebrospinal fluid of children with influenza-associated encephalopathy. Markedly elevated serum IL-6 may predict a poor outcome in these patients.[6] Some authors have suggested that underlying metabolic disorders or genetic susceptibility may play a role in the pathogenesis.[7]

Neurological complications (seizures, encephalopathy, encephalitis) associated with H1N1 have now been described from some countries.[1,2] The severity of the neurologic disease in most of these cases was discrete, with no deaths and no neurologic sequelae at discharge. Similarly, our patient presented a favorable outcome with full recovery within a few days. Recently, however, few cases have been reported with severe neurological symptoms and signs and dominant residual neurological sequelae.[8,9] It is noteworthy that both these patients were adults. There is a single case report of an adolescent with neuropsychiatric symptoms associated with novel influenza A (H1N1).[10]

EEG abnormalities were commonly observed in seasonal influenza-related encephalitis (86%) of patients in review of Amin *et al*.[4] Generalized or focal slowing on EEG can be a clue to the early diagnosis of encephalitis.[6] Three of the four patients in CDC report[1] had abnormal electroencephalograms. Our patient had focal slowing and sharp activity from the left centroparietal region

Kimura *et al* divided influenza-related brain changes into five categories based on the MR imaging and CT findings: normal (category 1), diffuse involvement of the cerebral cortex (category 2), diffuse brain edema (category 3), symmetric involvement of the thalamus (category 4), and focal encephalitis (category 5). Our patient’s CT findings are consistent with category 5 i.e. focal encephalitis. Lyon *et al*. reported CT and MR imaging findings in a 12-year-old girl infected with influenza A (H1N1) whose clinical course was complicated by acute necrotizing encephalopathy. The authors reported T2 hyperintensity and restricted diffusion in the thalami, cerebellar hemispheres and brain stem. Haktanir A. reported similar abnormalities and also bilateral perirolandic changes and diffuse meningeal enhancements.

Treatment of seasonal influenza in children with zanamivir and oseltamivir provided a more rapid resolution of symptoms and illness from 0.5 to 1.5 days.[8] No randomized, controlled studies have examined the antiviral treatment on influenza-related neurologic complications and it is not clear whether the treatment resulted in any clinical improvement or whether the neurologic symptoms were self-limited. Nonetheless, antiviral treatment should be initiated as soon as possible for any patient with neurologic symptoms related to H1N1 virus.

To the best of our knowledge, our patient is the first reported case of H1N1-related neurological manifestation from India. Clinicians should be alert to the potential for neurologic complications associated with H1N1.
